# Combined Influence of B-Cell Receptor Rearrangement and Somatic Hypermutation on B-Cell Class-Switch Fate in Health and in Chronic Lymphocytic Leukemia

**DOI:** 10.3389/fimmu.2018.01784

**Published:** 2018-08-10

**Authors:** Velislava N. Petrova, Luke Muir, Paul F. McKay, George S. Vassiliou, Kenneth G. C. Smith, Paul A. Lyons, Colin A. Russell, Carl A. Anderson, Paul Kellam, Rachael J. M. Bashford-Rogers

**Affiliations:** ^1^The Wellcome Sanger Institute, Hinxton, United Kingdom; ^2^Department of Medicine, Division of Infectious Diseases, Imperial College London, London, United Kingdom; ^3^Department of Medicine, University of Cambridge, Cambridge, United Kingdom; ^4^Department of Medical Microbiology, Academic Medical Center, University of Amsterdam, Amsterdam, Netherlands

**Keywords:** B-cell receptor seq, chronic lymphocytic leukemia, isotype switching, B cells, repertoire analysis

## Abstract

A diverse B-cell receptor (BCR) repertoire is required to bind a wide range of antigens. BCRs are generated through genetic recombination and can be diversified through somatic hypermutation (SHM) or class-switch recombination (CSR). Patterns of repertoire diversity can vary substantially between different health conditions. We use isotype-resolved BCR sequencing to compare B-cell evolution and class-switch fate in healthy individuals and in patients with chronic lymphocytic leukemia (CLL). We show that the patterns of SHM and CSR in B-cells from healthy individuals are distinct from CLL. We identify distinct properties of clonal expansion that lead to the generation of antibodies of different classes in healthy, malignant, and non-malignant CLL BCR repertoires. We further demonstrate that BCR diversity is affected by relationships between antibody variable and constant regions leading to isotype-specific signatures of variable gene usage. This study provides powerful insights into the mechanisms underlying the evolution of the adaptive immune responses in health and their aberration during disease.

## Introduction

The adaptive immune system possesses mechanisms for generating diverse repertoires of B-cell receptor (BCRs), which enable the specific recognition of a vast number of encountered antigens (estimated unique BCRs in an individual ≈10^11–13^) (1). While this breadth of antigen specificities ensures protection against a wide range of infectious diseases, any dysregulation in the balance between foreign- and self-antigen discrimination can lead to immunodeficiency, autoreactivity, or malignancy.

B-cell receptors can be expressed on the surface of B-cells or secreted as antibodies. Each BCR has an antigen-binding N-terminal variable region and a C-terminal constant region involved in effector functions. The generation of BCRs, each with different antigen specificities, involves several layers of genetic diversification in immunoglobulin heavy (*IGH*) and light chain (*IGL/IGK*) genes, which occur before and after antigen exposure. B-cells originate from hematopoietic stem cell progenitors in the bone marrow. They contain multiple distinct variable (V), diversity (D) (for the heavy chain only), joining (J), and constant (C) gene segments within the immunoglobulin gene loci (2, 3). These genes are highly polymorphic and constitute the basis for the germline BCR allelic diversity of an individual. During B-cell maturation, individual V, D, and J gene segments (only V and J in the light chain) undergo recombination forming the functional BCR protein. The germline allelic diversity, together with the combinatorial V-(D)-J diversity and imprecise joining of the V-(D)-J gene segments generates a highly diverse pre-immune (naïve) B-cell repertoire (1).

The additional diversity of mature BCR repertoires may be generated after exit from the bone marrow following antigen encounter. The binding of a BCR to an antigen, along with other auxiliary signals (e.g., from T-cells, cytokines, and TLR ligands) leads to B-cell activation, which is characterized by clonal expansion (4) and potential diversification of BCR molecules *via* somatic hypermutation (SHM) and class-switch recombination (CSR). SHM introduces mutations within the variable region of BCR which affects the binding affinity to antigen. Cells with high-affinity may be selected to expand further, a process that typically occurs in specialized structures known as germinal centers (GCs) (5).

Class-switch recombination involves the deletion of intervening DNA between constant genes within the *IGH* locus and results in the relocation of a constant region gene to the recombined VDJ portion of a BCR. The identity of the recombined constant region gene determines the BCR isotype (class) and the associated antibody effector functions. There are five main groups of BCR classes in humans, namely IgD, IgM, IgG1-4, IgA1-2, and IgE. The function and abundance of each antibody isotype varies throughout the body, and can trigger different immune responses to specific antigens by interaction with specific Fc receptor molecules (6–8). An increasing number of studies also attribute a direct role of the antibody isotype on its antigen-binding affinity by affecting antibody secondary structure (9, 10). These observations suggest that during antigen-driven clonal expansion, B-cells are selected not only based on their variable genes but also for the optimal combinations of variable genes and isotypes leading to successful antigen recognition and neutralization.

While the recognition of specific antigens is the major driver of SHM and class-switching in healthy B-cell repertoires, clonal evolution can also result from a malignant process of expansion of specific B-cell populations with or without antigen stimulation. CLL is an example of a B-cell malignancy characterized typically by the accumulation of clonally related CD19^+^CD5^+^IgM^+^IgD^+^ B-cells and constitutively active BCR signaling which plays a role in disease progression (11, 12).

These CLL B-cells can harbor mutated or unmutated *IGHV* genes, with the degree of SHM acting as a prognostic marker of disease outcome (13, 14). CLL clones from different individuals show stereotypical enrichments of certain *IGHV* genes [e.g., *IGHV3-21*, estimated to be ~30% of CLL cases (15, 16)] regardless of *IGHV* mutational status (17–20). There is still debate about whether this enriched *IGHV* gene usage is a result of a response to common antigens or a shared mechanism of clonal expansion driving the evolution of a malignant clone. The presence of highly expanded malignant clones that can undergo SHMs without class-switching questions the necessity for antigen-dependent stimulation and suggests that a different mode of clonal expansion can drive the evolution of CLL clones (21, 22). Antigen-independent cell-autonomous signaling has been proposed as a mechanism driving CLL malignancy and it shows a dependence on the specific sequence features of its variable genes (23). A better understanding of the mechanisms underlying the differences in B-cell clonal expansion in health and in malignancy requires a comprehensive characterization of the processes of SHM and CSR, and the resulting clonal selection that drive the generation of B-cell BCR diversity.

Sequencing BCR repertoires provides an opportunity for monitoring the evolution of B-cell responses by characterizing the sequence diversity of BCR genes. Multiple studies have already demonstrated the utility of sequence profiling of BCR repertoires for understanding adaptive immune responses in healthy individuals and in a range of clinical contexts (24–26). With advances in high-throughput sequencing and the ability to correct PCR amplification biases and sequencing errors through the inclusion of unique molecular identifier tagging (barcoding) (27), BCR sequencing has the potential to reliably quantify aspects of adaptive immune responses. However, the majority of the studies using BCR sequencing to characterize B-cell responses in health and disease focus on *IGHV-*(*D*)*-J* gene usages and SHM independently as a measure of diversity and clonal evolution of a B-cell repertoire (28, 29). These approaches have limited capacity to characterize the coupled interaction between SHM and CSR as two related processes underlying the evolution of B-cell responses.

Here, we developed an isotype-resolved barcoded BCR sequencing method to characterize the mutational processes driving the diversity of BCR repertoires in B-cells from peripheral blood of healthy individuals and individuals with CLL. We identify distinct properties of clonal expansion that lead to the generation of antibodies of different classes in healthy and malignant BCR repertoires. We further demonstrate that BCR diversity is affected by relationships between antibody variable and constant regions leading to isotype-specific signatures of variable gene usage.

## Materials and Methods

### Samples

Peripheral blood mononuclear cells (PBMCs) were isolated from 10 mL of whole blood from 19 healthy volunteers and 6 CLL patients using Ficoll gradients (GE Healthcare). Research was approved by the Wellcome Sanger Institute review boards and ethics committees (07/MRE05/44). Ethical approval for this study was obtained from the Cambridge Local Research Ethics Committee (reference numbers 04/023, 08/H0306/21, and 08/H0308/176) and Eastern NHS Multi Research Ethics Committee (07/MRE05/44), with informed consent obtained from all subjects enrolled. The FACS sorting and sequencing of the samples described in Figure S7 in Supplementary Material was approved by the MREC for Wales under REC number 12/WA/0196.

### FACS Sorting of B Memory Cell Subsets

Isolated PBMCs from a healthy adult volunteer were incubated with 100 µL Aqua Live/Dead viability dye (Thermo Scientific, UK) per 1 × 10^6^ cells for 20 min at room temperature in the dark. Viability dye was prepared by diluting 1:400 in PBS. Cells were then washed with PBS, and incubated with the pre-titrated B cell phenotyping panel shown in Table S5 for 30 min at room temperature in the dark. Following incubation, cells were washed with PBS and re-suspended in warm RPMI 1640 medium (Life Technologies, UK) supplemented with 100 U/mL penicillin (Life Technologies), 100 µg/mL streptomycin (Life Technologies), 2% FCS, and 2 mM l-glutamine (Life Technologies) (R2 medium). CD27^+^ memory B cells were sorted into four distinct populations based on IgM, IgD, and IgG expression (IgD^+^ IgM^+^, IgD^−^ IgM^+^, IgD^−^ IgM^−^ IgG^−^, and IgD^−^ IgM^−^ IgG^+^). Cells were sorted directly into 1.5 mL microcentrifuge tubes containing RPMI 1640 medium supplemented with 100 U/mL penicillin, 100 µg/mL streptomycin, 2 mM l-glutamine, and 10% FCS. Sorting was carried out using a BD FACSAria III and data analyzed using FlowJo software 9.7.5 (TreeStar, Ashland, OR, USA). Antibody panels used are provided in Table S5 in Supplementary Material.

### Comparison of Library Preparation Methods and Library Preparation for Isotype-Resolved Sequencing

RNA extraction was performed using RNeasy Mini Kit (Qiagen) according to manufacturer’s protocol. Reverse transcription (RT) was run as a 20 µL reaction with SuperScript^®^ III (Thermo Fisher) for 3′MPLX and 5′MPLX reactions and SuperScript^®^ II (Invitrogen™) for 5′RACE reactions. cDNA was cleaned-up with Agencourt AMPure XP beads in each of the three methods. Reagents for each RT step were divided in two mixes. For 5′RACE protocol Mix1 (RNA template, 12 µM reverse “oligo dT” primer, and nuclease-free water) was incubated for 1 min at 70°C and then immediately transferred on ice for 1 min. Mix2 (3.5 µL 5× FS buffer, 1 µL DTT (20 mM), 1 µL dNTP (10 mM), 1 µL RNaseOut^®^, and 1 µL Superscript II) was added and incubated at 42°C for 30 min. After 30 min, reactions were removed from the thermal cycler and 1 µL of “5′Oligo” (12 nM) was added. RT was resumed at 42°C for 90 min, followed by inactivation at 70°C for 10 min. Cleaned cDNA was amplified using KAPA Real-Time Library Amplification Kit (KAPA Biosystems) with 10 µM “5′universal” primer and 10 µM “JH”—*IGHJ*-gene primer and the following thermal cycling conditions: 1 step (95°C—5 min); 5 cycles (98°C—5 s; 72°C—2 min); 5 cycles (65°C—10 s, 72°C—2 min); 25 cycles (98°C—20sec, 60°C—1 min, 72°C—2 min); and 1 step (72°C—10 min). For 3′MPLX protocol Mix1 (RNA template, 12 µM reverse “JH_BC” primer, and nuclease-free water) was incubated for 1 min at 70°C and then immediately transferred on ice for 1 min. Mix2 [4 µL 5× FS buffer, 1 µL DTT (0.1 M), 1 µL dNTP (10 mM), and 1 µL Superscript III] was added and incubated at 50°C for 60 min followed by inactivation at 70°C for 15 min. Cleaned cDNA was amplified with non-barcoded V-gene multiplex primer mix (“VH1- to VH6-FR1” primers) (10 µM each forward primer) and “3′ universal” reverse primer (10 µM) using KAPA protocol and the thermal cycling conditions described above for the 5′RACE method. For the 5′MPLX RT step was carried out as for 3′MPLX method but with non-barcoded “JH” *IGHJ*-gene reverse primer. cDNA was cleaned up and amplified for 5 cycles with KAPA enzyme and barcoded forward V gene primer mix (“VH1- to VH6_FR1_BC”) (2.5 µM each primer) and non-barcoded “JH” *IGHJ*-gene reverse primer with the following thermal cycling conditions: 1 step (95°C—5 min); 5 cycles (98°C—20 s, 60°C—1 min, and 72°C—2 min); and 1 step (72°C—10 min). PCR product was cleaned-up and amplified further with “5′universal” forward primer (10 µM) and non-barcoded “JH” *IGHJ*-gene reverse primer (10 µM). Thermal cycling conditions were the same as for 3′MPLX method. The library preparation steps for isotype-resolved sequencing outlined in Figure S6 in Supplementary Material were identical as the ones used for the 3′MPLX method with the exception of the reverse primers used for the RT step. The reverse primers used for isotype-resolved sequencing were specific to the *IGHC* (constant region) genes for each BCR class. A mixture of these five reverse primers (labeled as “IGHA”, “IGHM”, “IGHD”, “IGHE”, and “IGHG”) was used for RT at a concentration of 10 uM per primer. Nucleotide sequences of all used primers described above are provided in Table S3 in Supplementary Material. Primers used for methods comparison are annotated as “3′MPLX”, “5′MPLX”, and “5′RACE” in the “Methods used” column of Table S3 in Supplementary Material. Primers used for isotype-resolved sequencing are annotated as “Isotype” in the “Methods used” column.

### Sequencing and Barcode Filtering

MiSeq libraries were prepared using Illumina protocols and sequenced using 300 bp paired-ended MiSeq (Illumina). Raw MiSeq reads were filtered for base quality (median Phred score > 34) using the QUASR program (http://sourceforge.net/projects/quasr/) (30). MiSeq forward and reverse reads were merged together if they contained identical overlapping region of >50 bp, or otherwise discarded. Universal barcoded regions were identified in reads and orientated to read from V-primer to the constant region primer. The barcoded region within each primer was identified and checked for conserved bases (i.e., the T’s in NNNNTNNNNTNNNNT sequence). The reads were checked for homology to the first 50 bp of the reference constant region genes from the IMGT (31) database by k-mer matching (where *k* = 10 bp). The closest matching constant region alleles were identified and information retained throughout the analysis. Primers and constant regions were trimmed from each sequence, and sequences were retained only if there was >80% sequence certainty between all sequences obtained with the same barcode, otherwise discarded. The error rate estimated from the data before barcode correction is 0.408% through calculation of the number of mismatches in sequences sharing the same barcode. Sequences without complete reading frames and non-immunoglobulin sequences were removed and only reads with significant similarity to reference *IGHV* and *IGHJ* genes from the IMGT database were retained using BLAST.

### Sequence Annotation and Network Analysis and Clustering

*IGHV* and *IGHJ* genes and mutational status were determined for each BCR using IMGT/V-QUEST (32). The network generation algorithm and network properties were calculated as previously described (33). Briefly, each vertex represents a unique sequence in which relative vertex size is proportional to the number of identical sequence reads after barcode correction. Edges are generated between vertices that differ by single-nucleotide, non-indel differences and clusters are collections of related, connected vertices.

### Calculation of Isotype Frequency, Complementarity Determining Region 3 (CDR3) Length, and Mutation Levels Across Cohorts

Isotype frequencies were calculated for each cohort as percentage of a unique *IGHV-D-J* regions per isotype as a proportion of all unique *IGHV-D-J* regions. Mean CDR3 lengths per unique *IGHV-D-J* region per isotype and median SHM levels (including silent and non-silent mutations) per unique *IGHV-D-J* region per isotype were calculated for each individual and compared across cohorts. The *p*-values of the difference in each of the metrics were assessed using Wicoxon rank-sum test. Only unique BCRs were included in all analyses of isotype frequency, CDR3 length, and mutation in *IGHV* gene to control for differential RNA per cell and thus reducing potential biases from differential RNA per cell, such as from plasmablasts.

### Separation of CLL and Non-CLL BCRs From CLL Patient BCR Repertoires

Non-malignant B-cell clones were defined as clones not containing BCRs with the following properties: (1) BCRs that are part of the largest CLL cluster, (2) BCRs with identical CDR3 regions as those defined in (1), and (3) BCRs with the same V-J combinations as those in part (1). The leukemic repertoire was defined as BCR sequences that are: (1) part of the largest CLL cluster and (2) BCRs with identical CDR3 regions as those defined in (1).

### Overlap Coefficients Between Isotypes

The overlap coefficient between any two isotypes, IgX and IgY, is defined as the relative frequencies of BCR reads with identical V-D-J regions but represented by only isotypes IgX and IgY. To account for differences in sequencing depth between samples, we performed 20 subsamples of 8,000 reads per sample generating a mean overlap coefficient value.

### Conditional Probability of a BCR Being Switched to a Given Isotype, Given Its Variable Gene Family Identity and Mutational Status

Conditional probabilities were calculated for *IGHV* genes represented by more than 100 reads (in total across samples). All conditional probabilities were calculated based on unique BCRs to control for differential RNA per cell and thus removing potential biases from differential RNA per cell, such as from plasmablasts.

fx(total repertoire) = total number of reads (filtered as described above)
*P(Mutated|VgeneX)* = *fx(VgeneX with 1 or more nucleotide mutations in V gene)/fx (VgeneX)*
*P(Unmutated|VgeneX)* = *fx(VgeneX with no mutations in V gene)/fx(VgeneX)*
*P(Switched|VgeneX,unmutated)* = *fx(VgeneX AND switched AND unmutated)/fx(Unmutated AND VgeneX)*
*P(Switched|VgeneX,mutated)* = *fx(VgeneX AND switched AND mutated)/fx(Mutated VgeneX)*

### Conditional Probability of a BCR Being Switched to a Given Isotype, Given Its Variable Gene Identity

Conditional probabilities were calculated for *IGHV* genes represented by more than 100 reads (in total across samples) and present in more than 5 samples from healthy PBMCs.
fx(total repertoire) = total number of reads (filtered as described above)
*P(Switched|VgeneX)* = *fx(VgeneX in any switched isotype)/fx(VgeneX)*
*P(IsotypeY|VgeneX, switched)* = *fx(VgeneX AND switched to Isotype Y)/fx(Switched AND VgeneX)*

### Calculation of Isotype-Specific *IGHV* and *IGHJ* Gene Profiles

Isotype-specific IGHV gene frequency profiles were calculated for each healthy individual based on the frequency (%) of an IGHV gene in the total number of reads associated with a given isotype. Only unique BCR sequences were considered. IGHV genes represented by less than 100 reads were excluded.

## Results

### Distinct Isotype Profiles in Healthy and Malignant BCR Repertoires

Accurately capturing the full complexity of BCR repertoires by high-throughput sequencing poses substantial technical challenges, including PCR and sequencing errors, biased transcript amplification and insufficient transcript capture efficiency, which can affect the estimation of true SHM rate and repertoire diversity (34–36). To ensure high accuracy of repertoire capture, we compared the performance of three methods for introducing molecular barcodes to individual BCRs during PCR amplification (see Supplementary Text in Datasheet S1 in Supplementary Material). RT primer-barcoding and multiplex *IGHV* gene amplification demonstrated minimal amplification bias and the highest sensitivity and reproducibility of repertoire characterization (Figures S1–S5 in Supplementary Material). We used this barcode incorporation approach as a basis of a RT-PCR strategy for amplification of all antibody classes simultaneously (Figures S6 in Supplementary Material). The isotype frequencies in each sorted B-cell population derived by BCR sequencing reliably reflected the surface expression of individual B-cell subsets (Figures S7 in Supplementary Material). Hence, we applied the isotype-specific BCR sequencing method on peripheral blood samples from 19 unrelated healthy individuals and from 6 CLL patients (Table S4 in Supplementary Material).

**Figure 1:**
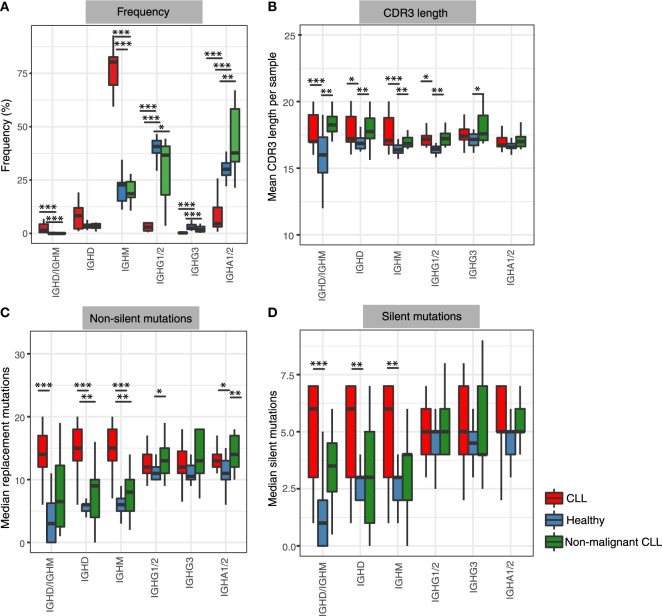
Frequency and mutational status of antibody isotypes in healthy and in leukemic repertoires. **(A)** Frequency of unique B-cell receptor (BCR) corresponding to each isotype across total CLL repertoires (red), healthy peripheral blood mononuclear cell repertoires (blue), and non-malignant CLL repertoires (excluding the major malignant clones) (green). Frequency of each isotype (sub)class is represented as a percentage of the unique BCRs corresponding to a specific isotype per individual. **(B)** Differences in complementarity determining region 3 (CDR3) amino acid lengths of unique BCRs corresponding to each isotype calculated as mean CDR3 length per isotype per sample. **(C)** Differences in non-silent mutations in *IGHV* genes of unique BCRs calculated as median non-silent mutation per isotype per sample. **(D)** Differences in silent mutations in IGHV genes of unique BCRs calculated as median silent mutation per isotype per sample. In all panels ***p-value < 0.0005; **p-value < 0.005; and *p-value < 0.05 (Wilcoxon rank-sum test of the respective metric in each panel compared between healthy and disease groups).

We first compared the isotype frequency distribution in healthy and leukemic repertoires. The isotype frequencies were calculated from the number of unique BCRs to control for differential RNA per cell and thus reducing potential biases from differential RNA per cell, such as from plasmablasts. The healthy BCR repertoires were dominated by IgM, IgA1/2, and IgG1/2 subtypes (Figure 1A), consistent with the predominance of naïve B-cells in peripheral blood (~64% of peripheral blood) as well as IgA^+^, IgG^+^, or IgM^+^ memory B-cells (~30% of peripheral blood) (37). The high percentages of BCRs of IgA1/2 and IgG1/2 isotypes may also represent plasmablast/plasma cell populations (38) which constitute a small proportion of the circulating B-cells [~2.1% of the peripheral blood (37)] but express high levels of BCR RNA (>1,000-fold higher per cell than naïve B-cells) and thus have a higher sampling and sequencing probability from a bulk PBMC population. By contrast, CLL repertoires were comprised of primarily unswitched (IgD/IgM) BCRs and significantly lower representation of IgA1/2 and IgG1/2 isotypes (*p*-value <0.0005, Figure 1A).

To investigate if the distinct isotype profile in CLL is driven by the malignant clonal expansion or a characteristic of the entire B-cell repertoire from CLL patients, we calculated the isotype frequencies of the CLL repertoires outside of the major malignant clone for each patient. To derive the non-malignant B-cell counterpart, we removed the expanded leukemic clone BCRs as well as all BCRs with *IGHV-J* gene combinations and complementarity determining region 3 (CDR3) sequences identical to this clone. The non-malignant CLL repertoire, which comprised between 1.5 and 71.3% (median of 4.6%) of the total CLL repertoires, showed IgD and IgM isotype frequencies similar to healthy repertoires, with a predominant expression of IgA1/2 and IgG1/2 (mean of 37%). Interestingly, IgA1/2 isotypes were present at significantly higher levels in non-malignant CLL repertoires compared to healthy (*p*-value <0.005). This overrepresentation of IgA1/2 isotypes in non-malignant CLL repertoires was not an artifact of the depletion of the *IGHV-J* genes associated with the leukemic clones as the enrichment of IgA1/2 was maintained (*p*-value <0.0005) even after the removal of the same *IGHV-J* gene combinations from the healthy repertoires (Figure S8 in Supplementary Material). Together, these show that the elevated levels of IgD and/or IgM BCRs is a feature of the malignant clones in CLL and the remaining BCR repertoire shows unaltered levels of unswitched BCRs but a skewed class-switch profile.

### CDR3 Lengths Are Distinct Between BCR Isotypes and Between Healthy and CLL Repertoires

We next examined CDR3 length distributions between healthy individuals and CLL patients. CDR3 is the most variable region of the antibody sequence, driven in part by the insertions and deletions of nucleotides between *IGHV, IGHD*, and *IGHJ* genes during recombinaiton, as well as *IGHV* gene editing. CLL patients exhibited increased CDR3 lengths compared to healthy controls in both IgD/M and IgG1/2 BCR subsets (Figure 1B). The propensity of longer CDR3 regions was also observed in the non-malignant B-cell counterpart of the CLL repertoires in both the unswitched (IgD and IgM) and IgG BCR subsets. This suggests that despite the maintenance of normal frequency of unswitched isotypes in the non-malignant B-cell clones in CLL patients, these repertoires exhibit a general skew to longer CDR3 lengths.

### Levels of SHM Are Distinct Between BCR Isotypes and Between Healthy and CLL Repertoires

Having demonstrated that both the malignant B-cell expansion and the non-malignant B-cell counterpart exhibit significant differences in isotype frequencies and CDR3 lengths compared to healthy individuals, we next investigated if BCR isotypes also differ in the degree of SHM in their *IGHV* genes. The number of SHMs within a BCR sequence relates to the degree of affinity maturation undergone by the corresponding B-cell clone, which in turn typically relates to the degree of antigen-exposure and activation experienced by the clone (39). We calculated the frequency of non-silent and silent SHMs per isotype to distinguish mutational processes that are likely to result in changes in the antigen-binding property of a BCR compared to those that would not alter the affinity to an antigen. The mean percentage frequencies of non-silent and silent mutations per BCR were significantly different between switched and unswitched isotypes in healthy repertoires (*p*-value <0.001) (Figures 1C,D). The BCRs with *IGHV-D-J* regions identified in both IgD and IgM isotypes (denoted “IgD/IgM”) showed the lowest mutation frequency in their *IGHV* genes (average non-silent and silent mutations: median of 2.6 and 1.1 mutations per BCR respectively). This is consistent with the large proportion of IgD/IgM BCRs originating from naïve B-cells in healthy repertoires, which by definition are antigen inexperienced and have not undergone SHM (37).

By contrast, the malignant CLL repertoires showed consistently high levels of SHM in both the unswitched and switched BCRs compared to healthy individuals. Interestingly, the non-malignant B-cell counterpart of the CLL repertoires also exhibited elevated levels of both non-silent and silent SHM compared to healthy controls in the IgD and IgM BCR subsets. This suggests that malignant clones can undergo substantial SHM without class-switching, while such high levels of SHM are primarily observed in class-switched clones in healthy and non-malignant BCR repertoires. IgA1/2^+^ non-malignant B-cells showed elevated non-silent mutations compared to healthy controls in addition to the elevated proportions of IgA1/2 (Figure 1A).

### Clonal Expansion, SHM, and Class-Switching Are Intrinsically Linked Processes in Healthy B-Cell Populations

**Figure 2:**
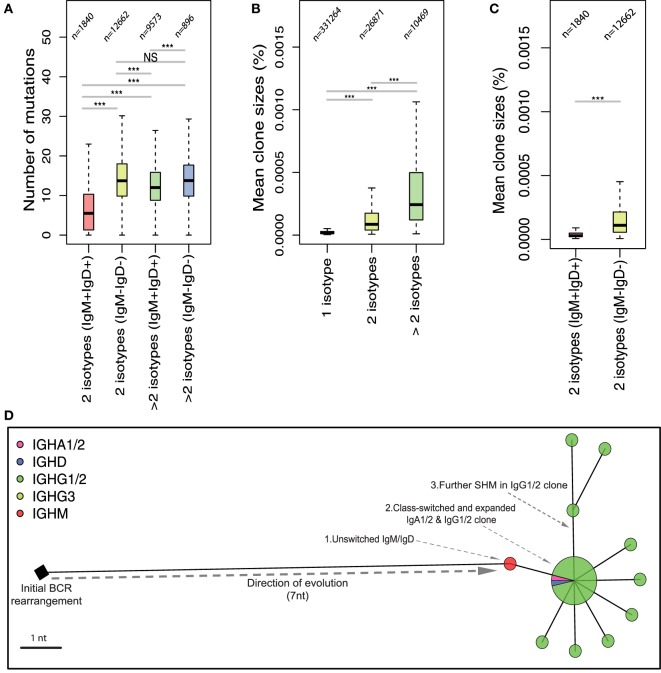
Relationship between somatic hypermutation and class-switching in the evolution of B-cell responses in healthy repertoires. Repertoires were clustered into networks of related B-cell receptors (BCRs) as previously described (33). **(A)** Boxplots of the mean number of mutations from germline in clones exhibiting 2 isotype classes or greater than 2 isotype classes that are either IgM^+^IgD^+^ or IgM^−^IgD^−^. The *y*-axis represents the number of nucleotide mutations in *IGHV* genes of BCRs associated with the respective number of isotypes. **(B)** Boxplots of the mean size of BCR clones formed by the respective number of isotypes shown on the *x*-axis as a proportion of total BCR reads. **(C)** Boxplots of the mean size of clones exhibiting 2 isotype classes that are either IgM^+^IgD^+^ or IgM^−^IgD^−^. Numbers above each boxplot in **(A–C)** represent the total number of clones considered for each isotype group. **(D)** A maximum parsimony tree of a class-switched BCR cluster in healthy repertoire representing an example of a clonal expansion following class-switching. ***p-value = 0.0005 and **p-value = 0.005 (Wilcoxon rank-sum test).

To investigate how the observed differences in isotype frequencies and *IGHV* mutational level relate to the processes of healthy versus malignant B-cell clonal evolution, we next characterized the relationship between mutation, class-switching, and clonal expansion in healthy and leukemic repertoires. We defined a B-cell clone as a group of BCRs related by single-nucleotide mutations (33), and compared the level of SHM from clones associated with different combinations of isotypes (Figure 2A). We show that the BCRs derived from clones exhibiting only IgM and IgD isotypes (2 isotypes IgM^+^IgD^+^) have low levels of SHM (mean of 5.205 nucleotide substitutions from closest germline *IGHV* gene), suggesting that these clones likely represent naïve B-cells or antigen-experienced (T-independent) B-cell clones that have not undergone an extensive GC reaction. Clones that are associated with 2 class-switched isotypes (2 isotypes IgM^−^IgD^−^) showed significantly higher levels of SHM (*p*-value <0.0005). Additional class-switching to more than 2 isotypes and loss of IgD and IgM clones was associated with a further increase in the mutational level of BCR variable genes.

In addition to the higher levels of SHM, class-switching was also associated with clonal expansion. Clones that have undergone class-switch events to more than one isotype showed a significantly increased mean clone size compared to single-isotype clones (Figure 2B). Loss of IgM and IgD expression during clonal evolution was further associated with an increase in clone size (Figure 2C). As an example of the combined processes of clonal expansion, SHM and CSR, we generated a phylogenetic tree of a representative clone from one healthy individual. This demonstrates the stepwise evolution of B-cell BCR from the initial BCR rearrangement to B-cells expressing BCRs containing increasing numbers of somatic mutations and subsequent class-switching. Class-switched B-cells undergo further clonal expansion and somatic mutation suggesting ongoing activation of the class-switched BCRs (Figure 2D).

### Malignant CLL B-Cells Can Undergo Class-Switching After Which Limited Clonal Diversification Occurs

**Figure 3:**
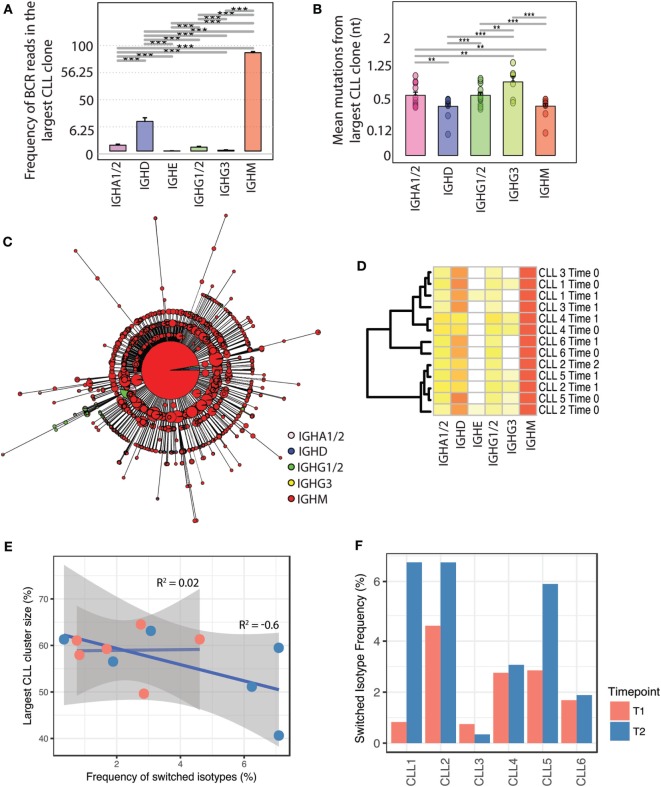
Relationship between somatic hypermutation, class-switching, and clonal expansion in the evolution of malignant B-cell clones. **(A)** Isotype composition of the largest CLL clone across all leukemic individuals. Isotype frequency was calculated as the percentage of reads in the CLL clone corresponding to a given isotype, on a square-root scale to distinguish between low frequency isotypes. **(B)** Number of mutated nucleotides in *IGHV* genes of B-cell receptors (BCRs) of different isotypes compared to the *IGHV* gene of the major leukemic CLL clone for each sample. **(C)** Unrooted phylogenetic tree of an example class-switched CLL clone. Each tree tip represents a unique BCR sequence, the pie charts represent the proportion of isotypes per unique BCR, and size corresponds to the proportion of BCRs represented by that sequence (square-root scale for visualization). **(D)** Heatmap of isotype frequency in total CLL repertoires of the six leukemic patients across timepoints of longitudinal sampling (see Table S4 in Supplementary Material). **(E)** Relationship between degree of class-switching and CLL clone size; the *x*-axis represents the total frequency of switched BCRs in the malignant CLL clone for each individual (represented as a dot). The *y*-axis reflects the size of the major CLL clone per individual shown as number of BCRs per individual contributing to the largest CLL clone (as a percentage of the total BCR repertoire). The correlation coefficient labels represent the correlations between percentage switched isotype per largest CLL clone and size of the largest clone for the two sampling points. **(F)** Frequency of switched BCR in the CLL malignant clones of each patient calculated for the two sampling points (T1 and T2) per sample. For sampling point details, see Table S4 in Supplementary Material.

While an increase in BCR clone size was characteristic of class-switched BCRs in healthy repertoires, the malignant clones in CLL patients comprised primarily of IgD and IgM BCRs, constituting on average total of 83.6% of BCRs across individuals. The remaining BCRs were derived from class-switched B-cells which were detected in all CLL patients and comprised between 0.042 and 2.72% of the BCR sequences in the malignant clone of each individual (Figure 3A). The BCRs associated with class-switched isotypes within the CLL clones (IgA1/2, IgE, IgG1/2, and IgG3) had a greater number of SHMs away from the central CLL BCR in all CLL patients sampled compared to IgM/D, suggesting a coupled process of SHM and B-cell class-switching in the malignant clone (Figure 3B).

Phylogenetic analysis of CLL clones (i.e., the population of related malignant B-cells) demonstrate that the majority of BCRs correspond to a dominant unswitched BCR sequence (Figure 3C). Indeed, sub-clonal diversity is generated through both SHM and CSR, resulting in CLL sub-clones comprising of B-cells exhibiting BCR variants of the dominant CLL BCR. Despite the presence of class-switching within the expanded malignant clones, class-switched clones do not dominate the subsequent clone progeny. The lack of further expansion of class-switched leukemic clones was maintained across multiple timepoints of sampling, and isotype frequencies were consistent between patients (Figure 3D). In contrast to healthy B-cell clones, class-switching in malignant B-cells was not associated with sub-clonal expansion and the frequency of class-switched BCRs showed poor correlation (*R*^2^ = 0.2, *p*-value = 0.96) with the size of the major CLL clone at the first time-point of sampling (Figure 3E). The lack of significant correlation between the degree of class-switching and level of SHM at the second point of sampling (*R*^2^ = −0.6, *p*-value = 0.2) further suggests that the increase in the size of CLL clones does not result from expansion of class-switched B-cells. At the second time-point, the contribution of class-switched isotypes to the major leukemic clone varied between 0.34 and 7.9% across the 6 CLL patients but did not show a consistent change over the two sampling time points (Figure 3F).

### Sequential CSR Frequencies Are Distinct Between Healthy and CLL B-Cell Repertoires

**Figure 4:**
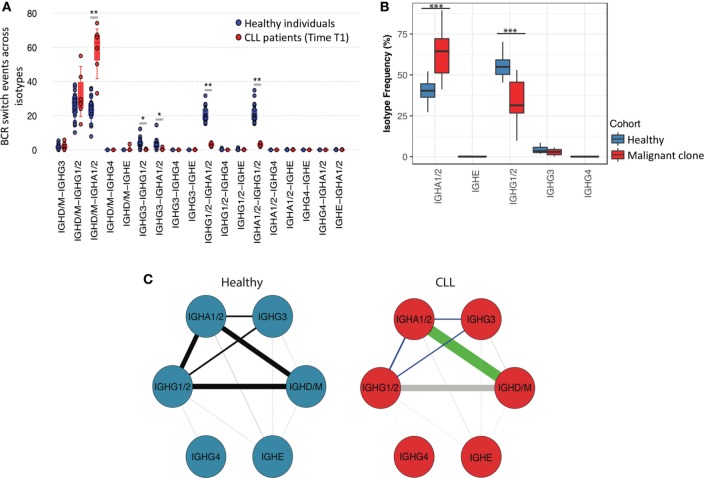
Sequential class-switching and isotype co-evolution in healthy and in malignant B-cell clones. **(A)** Boxplots of the proportion of observed class-switch events [B-cell receptor (BCRs) with identical *IGHV-D-J* regions but different isotypes] between isotypes for healthy individuals and CLL patients. This was calculated as the number of unique BCRs shared between any two isotypes after accounting for read depth through subsampling (see Materials and Methods), normalized to the total number of events. **(B)** Relative proportional frequencies of different isotype classes in healthy repertoires and in malignant clones across CLL patients. Malignant clones were derived for each CLL sample by removing the non-malignant component of the repertoire (i.e., the BCRs with VJ genes and complementarity determining region 3 amino acid regions different from the most frequent BCR per sample) from the total CLL repertoire. **(C)** Representative networks of the relative class-switch event frequencies between pairs of isotypes calculated as described in **(A)**. The thickness of the lines represents the mean relative class-switch event frequencies calculated as in **(A)**, and the green and light blue lines connect isotypes that exhibit significantly higher (green) or lower (light blue) overlap probability in CLL repertoires. The gray lines in the “CLL” panel represent combinations of isotypes with non-significant (*p*-value > 0.05) difference in the frequency of class-switch events compared to healthy repertoires.

The process of class-switching generates daughter B-cells with identical *IGHV-J* regions but distinct isotypes, producing antibodies enabling the activation of different effector mechanisms. Therefore, antibodies with identical variable genes but different isotypes represent the evolution of a B-cell clone which had undergone multiple class-switch events. To investigate how antibodies of different isotypes co-evolve in healthy versus malignant BCR repertoires, we next calculated the probabilities that a given BCR *IGHV*-*J* (antigen binding) region will be shared by any two isotypes, termed isotype clonal overlap. Here, we account for the differences in read depth between samples through subsampling 20× times each B-cell repertoire to a fixed depth (8,000 reads) and determining the mean number of BCRs shared between pairs of isotypes. Due to the sequencing of IgM-associated reads from IgD transcripts resulting from alternate splicing, these isotypes were analyzed together. In healthy individuals, the highest isotype clonal overlaps were between IgD/M and IgA1/2 or IgD/M and IgG1/2 classes (Figure 4A), suggesting that these are common switching transitions, and in agreement with a previous study (40). By contrast, CLL repertoires exhibited the highest isotype clonal overlap between IgD/M and IgA1/2, which was significantly higher than in healthy individuals (*p*-value < 0.005), indicating an increase in direct class-switching events to IgA1/2 in CLL. Indeed, a comparison of the relative frequencies of class-switched isotypes (i.e., excluding IgD/M BCRs) showed a significant enrichment of IgA1/2 isotypes and a reduced relative frequency of IgG1/2 in malignant CLL clones compared to healthy repertoires (*p*-value < 0.005) (Figure 4B). This suggests that the probability of class-switching to any isotype is dependent on the nature of the B-cell clone (i.e., malignant versus healthy) and despite the different genetics, signaling, and microenvironment between individual CLL cases, there is a consistent and significant bias in the fate of malignant B-cell clones to IgA1/2 isotypes.

The isotype clonal overlap between switched IgA1/2, IgG1/2, and IgG3 was significantly reduced compared to healthy individuals (*p*-values <0.005) (Figure 4C), suggesting that sequential class-switching events are rare in CLL. This is consistent with the lack of further clonal diversification of CLL B-cells following an initial class-switch. Together, these findings demonstrate that the evolution of the malignant CLL clone is distinct from that seen in antigen-driven clonal class-switching in healthy individuals, and that class-switching is not inherently linked to the clonal expansion and diversification of the B-cell clone.

### Distinct Profiles of *IGHV-IGHJ* Gene Usage Across BCR Isotypes

**Figure 5:**
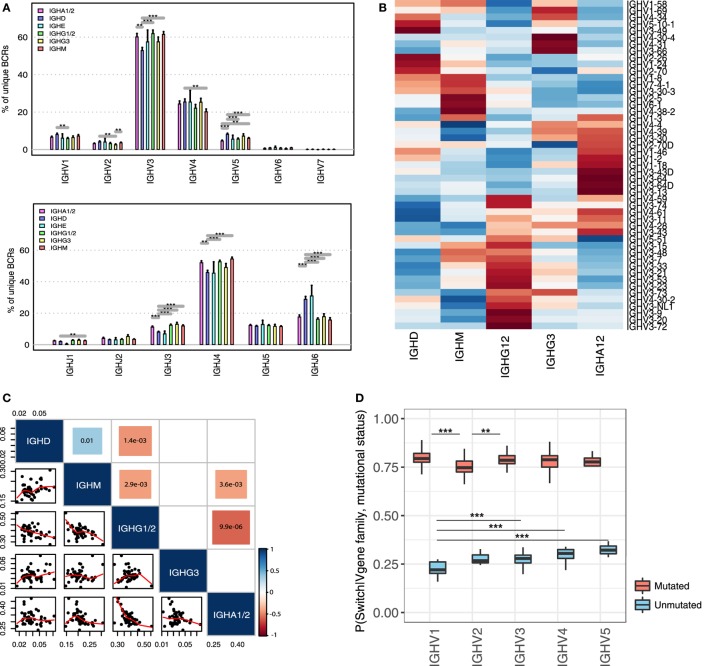
Role of somatic hypermutation and variable gene identity in the class-switch fate of healthy B-cell clones. **(A)** Differences in *IGHV* (upper panel) and *IGHJ* (lower panel) gene family usages between different isotype classes in healthy individuals. Frequencies of individual *IGHV*-J gene families are calculated as percentage of unique B-cell receptor (BCR) reads from each *IGHV* or *IGHJ* gene family associated with the respective isotype. **(B)** Heatmap of *IGHV* gene frequencies across isotypes. Individual values are scaled by row, key represents row-*Z* score and distance matrix between *IGHV* genes was generated using Euclidian distance. Only *IGHV* genes with >100 reads were considered. **(C)** Correlation of *IGHV* gene frequencies across isotypes. Only unique BCR sequences were considered and *IGHV* genes represented by less than 100 reads were excluded. The size and the color of the squares represent the absolute value and the direction of the correlation, respectively. The values in the colored squares represent the *p*-value of the significant positive or negative correlations across isotypes. Blank squares represent non-significant correlations (*p-*value > 0.05). The scatterplot matrix represents the frequencies of individual *IGHV* genes in the respective isotypes. Trendlines are shown in red. **(D)** Conditional class-switch probabilities of BCRs given in their *IGHV* gene identity and mutational status. Boxplots represent the variance of conditional probabilities of individual *IGHV* genes from the respective *IGHV* gene families. Only *IGHV* genes with >100 reads in both mutated and unmutated state were considered. ***p-value < 0.0005; **p-value < 0.001; and *p-value < 0.05 (Wilcoxon rank-sum test).

Although B-cell clones can switch to multiple isotypes during clonal expansion, antibodies of different classes are adapted to function in different anatomical regions and to generate antibodies capable of eliciting specific effector mechanisms (7). We showed that the processes of SHM and class-switching are intrinsically linked during the evolution of healthy B-cell clones. This suggests that the immune response to an antigen is likely to be dominated not just by antibodies with antigen-specific variable genes, but by those that also have switched to an isotype with appropriate effector functions. Therefore, we hypothesized that the overall composition of the antigen-binding regions associated with each isotype will be distinct, reflecting the binding of different sets of antigens in the context of different co-stimulatory signals. Indeed, we show significant differences in proportional *IGHV* and *IGHJ* gene family usage between isotypes across the 19 healthy individuals (Figure 5A). For example, even though, *IGHV3* and *IGHJ4* were the most abundant genes across all isotypes, *IGHV3* was significantly more common in IgA1/2 than IgD, whereas *IGHV5* was more frequently observed in IgD than in IgA1/2. Each isotype class exhibited a distinct *IGHV* gene expression signature when compared to unmutated IgD/IgM, suggestive that certain *IGHV-*constant region (*IGHC*) combinations are maintained or favored in healthy B-cell repertoires following antigen exposure (2 × 2 Chi-squared test of non-randomness: *p*-values: IgA1/2—0.01; IgE—<2.2e^−16^; IgG3—<2.2e^−16^; IgG1/2—0.0156; and IgG4—<2.2e^−16^). This preferential use of specific *IGHV* genes in specific (sub)classes was further demonstrated by the observed isotype-specific *IGHV* gene usage patterns (Figure 5B) and the significant negative correlation between IgG1/2 and IgA1/2 and unswitched IgM isotypes (Figure 5C). A positive correlation in *IGHV* gene usage was only detected between IgD and IgM isotypes, likely reflecting the presence of IgD^+^IgM^+^ B-cells, such as naïve B-cells that are the predominant B-cell type in peripheral blood.

### The Probability of a BCR to Be Switched to a Given Isotype Is Dependent on *IGHV* and *IGHJ* Gene Usage and Level of SHM

Considering the observed isotype-specific *IGHV* gene profiles across individuals, we next explored the role of *IGHV* gene identity in the class-switch fate of a B-cell clone. First, we tested the probability of a BCR being class-switched given its *IGHV* gene identity, *P* (switched | *IGHV* gene). This was calculated for all BCRs representing *IGHV* genes supported by more than 100 reads and present in more than 5 individuals. We used the variable gene identity and the conditional class-switch probability as predictor and outcome variables in a one-way ANOVA model and identified a significant effect of *IGHV* gene identity on the probability of a BCR to undergo class-switching (*p*-value: 3.759e^−06^). This suggests that different V genes have distinct probabilities of being present in a class-switched form in a healthy B-cell repertoire.

Since we showed that class-switching is associated with increasing level of SHM, we then investigated the effect of the mutational status of a BCR from a given *IGHV* gene family on its probability to be class-switched (Figure 5D). As expected, we found that across all *IGHV* gene families, BCRs have an increased probability of class-switch in a mutated form (*p*-value <0.005). In addition, there were significant differences in class-switch probability given the mutational state and *IGHV* gene family. The *IGHV1* gene family had a significantly reduced probability of class-switching in an unmutated form compared to *IGHV3, IGHV4*, and *IGHV5* gene families, while having the highest probability of class-switching in a mutated form. The lowest probability of class-switching after SHM was associated with the *IGHV2* gene family. Using a two-way ANOVA model, we next tested if the relationship between the mutational status of a BCR and its *IGHV* gene identity was associated with the class-switch fate of a B-cell. The degree of SHM and *IGHV* gene identity of a BCR individually acted as significant correlates of class-switch fate of a BCR (*p*-value <2 × 10^−16^). The probability of a clone existing in a class-switched state was also dependent on the relationship between *IGHV* gene and mutational status, with a significant interaction between these two factors (*p*-value = 0.0238). This suggests that the *IGHV* gene context in which mutation occurs may also be important for BCR clone evolution and class-switching.

Together, these data suggest that the class-switch status of a B-cell is influenced by the *IGHV* gene identity and mutational status of its BCR, which leads to the observed isotype-specific profiles of *IGHV* gene usage conserved across individuals.

## Discussion

The sequencing of the genetic diversity of immune receptor repertoires enables the characterization of B-cell fate. The combined capture of the diversity of variable genes together with the isotype usage of B-cell populations provides a comprehensive picture of the evolution of B-cell responses contributing to specific immune phenotypes. Here, we use isotype-resolved BCR sequencing to investigate the processes of B-cell clonal evolution that lead to the generation of antibodies of different classes in health and in leukemia. This method provides a platform for interrogating clonal evolution and functional diversification from B-cell samples in *in vivo*. Healthy B-cell repertoires demonstrate highly conserved patterns of isotype usage between unrelated individuals, with the dominance of IgM, IgG1/2, and IgA1/2 isotypes, in agreement with *Horns* et al. (40). The phenotype of a B-cell is reflected in the isotype and level of SHM of its BCR. For example, naïve B-cells express IgD and IgM BCRs with no SHM. Clones associated with class-switched isotypes are associated with higher levels of SHM as well as clonal expansion, thus providing a genetic indicator of B-cell clonal development and maturation.

By contrast, CLL B-cell repertoires are dominated by unswitched BCRs, which show significantly increased levels of SHM compared to healthy IgD or IgM antibody classes, suggesting a pattern of SHM without class-switching. This difference from clonal expansion in healthy B-cells demonstrates that the generation of BCR diversity follows different mechanisms in malignant B-cells. Interestingly, the high level of unswitched BCRs is not a feature of the entire CLL patient B-cell repertoire, but is restricted to the malignant clones, with the remaining BCR clones showing levels of unswitched isotypes similar to healthy individuals. However, the non-malignant CLL B-cell repertoire was found to be distinct from healthy individuals with increased frequency and higher SHM levels in IgA1/2 isotypes. Indeed, external signals to B-cells from the microenvironment in leukemia patients have been shown to be different from those in healthy individuals with significant contributions to B-cell survival and disease progression in CLL (41, 42). Our data are in agreement with perturbed environmental signals leading to differences in B-cell class-switch fate in the non-malignant CLL B-cell and the resulting different isotype profile.

In addition to the distinct isotype frequencies across leukemic and healthy repertoires, we showed a shift to longer CDR3 lengths in both the malignant and non-malignant CLL B-cell repertoire. Longer CDR3 lengths have been implicated in reactivity to self-antigens (43). Thus, the observed increase in CDR3 length in both switched and unswitched isotypes in the non-malignant CLL B-cell repertoires may correspond to altered tolerance mechanisms in CLL patients driving the increased propensity for autoimmune manifestations in these patients (44).

We further demonstrated that healthy and malignant B-cell clones follow distinct patterns of class-switching and clonal expansion. Clonal expansion in healthy repertoires is intrinsically linked to the processes of somatic mutation and class-switching. B-cells with mutated BCRs can class-switch to generate a population of B-cells of more than one isotype which are increased in SHM and undergo further clonal expansion. This pattern of clonal evolution is consistent with antigen-driven B-cell maturation with selection acting on BCRs with an optimal combination of specific variable genes and class-switched isotypes. B-cells from CLL clones also underwent class-switching in all six patients tested, which is in agreement with Efremov et al. demonstrating that CLL associated with IgA and IgG transcripts can be identified in CLL patients (21). We further identified that class-switching to IgE in leukemic repertoires. Moreover, we showed that malignant clones from different individuals preferentially switched to IgA1/2, suggesting a common feature of clonal evolution between patients. We also show that CLL clones exhibit distinct patterns of class-switching from healthy B-cells. This is consistent with a different mode of B-cell signaling, regulation, and/or selection within these malignant B-cell populations (45), which were maintained over the 1–9 months of sampling within these patients. While class-switched malignant B-cells were shown to be able to subsequently somatically hypermutate further, these class-switched sub-clones did not undergo significant levels of clonal expansion and subsequent class-switching as observed in healthy B-cell clones. Given that the proportion of class-switched BCRs per CLL clone did not significantly change over time, this suggests a steady state process of class-switching, where the resulting B-cells exhibited limited clonal expansion and diversification. Even though this is a limited number of patients assessed, all patients exhibited similar class-switching patterns despite representing different disease stages (Table S5 in Supplementary Material). This current work, therefore, lays the foundation for studying larger cohorts of CLL patients to understand the prognostic significance of this process.

We showed that somatic mutation in the variable genes and class-switching are intrinsically linked in the process of clonal expansion of B-cells in both healthy and CLL individuals. Given the distinct functions and distributions of antibody isotypes and their Fc receptors across anatomical locations, we investigated the relationship between the genetics of the antigen-binding region and the isotype fate of a B-cell clone. Antibody isotype classes showed profiles of *IGHV* gene usage and mutational status which were conserved across unrelated healthy individuals. The high level of conservation of these isotype-specific signatures of variable gene usage suggests an important relationship between the variable gene identity of a B-cell clone and its probability to populate a specific class-switched state in peripheral blood. The unmutated IgD/M BCRs are enriched for naïve B-cells which will have gone through central tolerance checkpoints, whereas the mutated and/or class-switched BCRs will have undergone additional peripheral tolerance checkpoints, such as antigen-driven clonal expansion and diversification. From these data, it is not possible to determine at what stage the variable gene identity affects the cell fate: whether this is imprinted within the bone marrow during the earliest stages of B-cell selection, such as through differences in B-cell anergy, or if class-switch fate is shaped during affinity maturation in the GC. A previous study has shown a high concordance of class-switch fate between sister B-cells *in vitro* (40) (same *IGHV-D-J* and closely related through SHM), but it was not clear whether cell fate concordance was due to BCR specificity or other mechanisms such as epigenetics. Here, we show that unrelated B-cell clones with the same *IGHV* gene usages also have significant concordance of cell fate compared to B-cell clones with distinct *IGHV* genes, suggesting that there is a strong variable gene effect on B-cell fate.

The observed isotype-specific patterns of variable gene usage in our study were also affected by the degree of mutation of variable genes—consistent with a model of B-cell class-switch fate linked to BCR specificity. The significant interaction between *IGHV* gene identity and mutational status on the probability of a clone existing in a class-switched state suggests that B-cell fate is dependent on both pre-antigen and post-antigen diversification processes, which are highly skewed in the context of CLL.

Our data are in agreement of a model in which the early class-switch decisions of a B-cell upon antigen stimulation have a degree of variable gene dependency which can be later revised by antigen-driven SHM and cytokine stimulation leading to class-switched clones. Such a role of variable gene status is interesting in the context of recent work by Greiff et al. (46) which describes a high degree of pre-determination of BCR repertoires and suggests that a specific genetic substructure underlies the diversity of BCR repertoires and can be used as a predictor of B-cell evolution. In the light of our data, this substructure may in part be formed by *IGHV* gene preference for certain isotypes.

A drawback of sequencing bulk PBMC populations is the potential biases from differential RNA levels per cell, such as from plasmablasts or plasma cells. This has been an issue in all previous RNA BCR sequencing studies that do not perform initial sorting of different B cell subsets. Importantly, we have normalized the BCR data through counting unique *IGHV-D-J* regions, thus controlling for differential RNA per cell. This normalization step corrected the overweight of the IGHV-D-J sequences expressed by plasmablasts and plasma cells but does not eliminate the relative contribution of BCR sequences form these subsets to the overall analysis. This is because the sampling probability of BCR transcripts from naïve B-cells with lower RNA expression means that there is an over-representation of BCRs from non-naïve B-cells, particularly plasmablasts and plasma cells. However, plasmablasts and plasma cells are the significant effector B-cell populations that are of particular interest in both health and disease. Therefore, sequencing the BCR transcripts from these rare cell types together with BCRs from naïve and memory B-cell populations provides us with important information about clonal expansion and final class-switching destination. Here, we demonstrate that isotype-resolved BCR sequencing can provide powerful insights into the mechanisms underlying the evolution of the adaptive immune responses in health and their aberration during disease.

## Data Access

The IgH sequences discussed can be found under Study Accession number EGAS00001002634 and EGAS00001002633 in the European Genome-Phenome Archive (EGA). Individual accession numbers are provided in Tables S1 and S2 in Supplementary Material. As the immunoglobulin sequences constitute human data, they are stored under managed data access according to the Wellcome Trust data release policy. Access to these samples must be requested from the Data Access Committee (DAC), whose contact details can be found on the EGA study page, accessible through the study accession numbers or by emailing datasharing@sanger.ac.uk. The requester will be required to sign a data access agreement, which is in place to protect the identity of the sample donor *via* a managed access system.

## Ethics Statement

Research was approved by the Wellcome Sanger Institute review boards and ethics committees (07/MRE05/44). Ethical approval for this study was obtained from the Cambridge Local Research Ethics Committee (reference numbers 04/023, 08/H0306/21, and 08/H0308/176) and Eastern NHS Multi Research Ethics Committee (07/MRE05/44), with informed consent obtained from all subjects enrolled.

## Author Contributions

VP designed, coordinated, and performed experiments, analyzed data, and wrote the paper. LM designed and performed some of the FACS sorting experiments and gave comments to the paper. PM designed and performed FACS sorting and gave comments to the paper. GV advised on experiments. KS designed the experiments and gave comments to the paper. PL advised on experiments and gave comments to the paper. CR contributed to the analysis and gave comments to the paper. CA contributed to the analysis and wrote the paper. PK designed and supervised the experiments and wrote the paper. RB-R designed and performed experiments, analyzed data, and wrote the paper.

## Conflict of Interest Statement

GV is a consultant for Kymab and receives an educational grant from Celgene. RB-R is a consultant for VHSquared. The remaining authors declare that the research was conducted in the absence of any commercial or financial relationships that could be construed as a potential conflict of interest.
