# Supplementary material for: Combined Influence of B-Cell Receptor Rearrangement and Somatic Hypermutation on B-Cell Class-Switch Fate in Health and in Chronic Lymphocytic Leukemia
Source: Front Immunol. 2018 Aug 10;9:1784. doi: 10.3389/fimmu.2018.01784 (PMC6095981; doi:10.3389/fimmu.2018.01784)
Supplement: Supplementary file 1 [file Data_Sheet_1.pdf]

Supplemental

1. Supplementary text

2. Supplementary tables

3. Supplementary figures - Provided as a separate file – “Data sheet 2.pdf”

1. Supplementary text

Comparison of barcode incorporation methods for error-corrected BCR sequencing

To ensure the accurate representation of BCR repertoires, we compared the performance of three methods for amplification and molecular barcoding of individual BCRs (3'multiplexing (3'MPLX), 5'multiplex (5'MPLX) and 5' Rapid Amplification of cDNA Ends (5'RACE), (See 'Methods' section in main text) **Supplementary figure S1**) in either highly diverse healthy repertoires derived from peripheral blood mononuclear cells (PBMCs) or in clonal lymphoblastoid cell line (LCL) samples (**Table S1**). We assessed the reproducibility of measures of BCR diversity across biological and technical replicates (**Supplementary figure S2**) and the sensitivity of each method for capturing rare B-cell clonotypes (V_HJ_H gene combinations).

Reverse primer barcoding during cDNA preparation and subsequent PCR amplification ('3'MPLX') provided the most reproducible method for measuring immunoglobulin variable gene frequencies with a mean correlation coefficient of R^2 = 0.9 between biological and technical replicates (**Supplementary figure S3**) and ability to re-capture at least once 30% of the unique BCR clonotypes from each sample across biological and technical replicates (**Table S2**). The 3'MPLX method also demonstrated the highest sensitivity of repertoire capture with detection of over 250 different IGHV-J combinations across a dynamic range of frequencies in the PBMC samples. In contrast, despite the use of equal amount of starting RNA and a lack of significant differences in read depth across methods (p-value > 0.05, Wilcoxon rank-sum test) the 5'RACE and 5'MPLX approaches showed a reduced breadth of the captured IGHV-J gene combination (**Supplementary figure S4**).

Comparison of the network structures derived for identical samples across the three amplification strategies showed that the differences in the method-specific diversity metrics result from substantial differences in the degree of introduced amplification biases. The 3'MPLX method captured between 9-90x more unique RNA molecules from the same amount of starting RNA and provided least amplification bias compared to the 5'MPLX and 5'RACE methods which exhibited artificially high clonality before barcode filtering (**Supplementary figure S5**). Considering the high sensitivity and minimal amplification bias observed in the 3'MPLX, we adopted this library preparation strategy as the basis of isotype-resolved BCR sequencing approach (**Supplementary figure S6).** Using five subsets of FACS sorted B memory cell populations we demonstrated that the BCR repertoires derived via an RT-PCR reaction with a mixture of isotype-specific primers reliably reflect the surface expression of BCR isotypes (**Supplementary figure S7).**

2. Supplementary tables

Table S1. Read processing and comparison of primer barcoding methods

Sample[1]	Raw reads (Fwd)	Raw reads (Rev)	Joined reads	Primer matched reads	Reads with ORF	Unique RNA molecules	Accession number
H1_a_3MPLX	543808	523060	403918	30199	30372	27863	EGAN00001588508
H1_a_5MPLX	176177	173523	112792	1631	1484	1384	EGAN00001588509
H1_a_5RACE	611997	617202	111029	3186	1300	1176	EGAN00001588510
H1_b_3MPLX	200119	104411	82730	31806	30719	18858	EGAN00001588514
H1_b_5MPLX	181880	105941	79652	573	553	312	EGAN00001588515
H1_b_5RACE	169730	127564	31202	6019	3485	3397	EGAN00001588516
H1_c_3MPLX	379587	228775	144743	63556	60689	49645	EGAN00001588523
H1_c_5MPLX	353715	231557	167611	693	663	303	EGAN00001588524
H1_c_5RACE	317716	256045	49557	10285	4985	4897	EGAN00001588525
H1_d_3MPLX	382270	230096	138326	61804	58843	47194	EGAN00001588526

H1_d_5MPLX	342769	195067	115877	853	813	445	EGAN00001588527
H1_d_5RACE	342844	289558	60133	10357	4067	3968	EGAN00001588528
H2_3MPLX	186565	102495	77076	30991	29644	25742	EGAN00001588517
H2_5MPLX	203347	118624	69636	237	223	123	EGAN00001588518
H2_5RACE	191504	154431	41358	3891	1480	1421	EGAN00001588519
H3_3MPLX	182958	103150	77521	31429	30017	27484	EGAN00001588520
H3_5MPLX	178516	103805	78254	408	362	177	EGAN00001588521
H3_5RACE	171018	137021	39307	4913	2322	2238	EGAN00001588522
LCL1_3MPLX	191009	84295	66836	34801	33161	6648	ERS939080
LCL1_5MPLX	177650	137102	83503	297	259	92	ERS939081
LCL1_5RACE	166344	128303	43528	7604	4040	3086	ERS939082
LCL2_3MPLX	633249	291988	210576	36538	28834	3804	ERS939077
LCL2_5MPLX	175653	96821	54327	251	54	25	ERS939078
LCL2_5RACE	264204	226759	49757	10067	3652	2651	ERS939079
LCL3_3MPLX	194448	108681	89420	62429	61469	5477	ERS939074
LCL3_5MPLX	167039	96877	60067	361	344	101	ERS939075
LCL3_5RACE	173134	144287	71538	12085	6123	2517	ERS939076
LCL4_3MPLX	1008500	722194	514525	23945	23010	2093	ERS939071
LCL4_5MPLX	182146	71326	51533	1792	1704	407	ERS939072
LCL4_5RACE	182776	155343	34395	12276	3867	2854	ERS939073

[1] H1_a to H1_d represent the same healthy sample analysed across PBMC/RNA/PCR/MiSeq replicates for reproducibility analysis (see ‘Methods’)

Table S2 Reproducibility of unique BCR frequency estimates across biological and technical replicates

Reproducibility[1]	3MPLX[2]	5RACE[3]	5MPLX[4]
1	0.72507	0.95693	0.93510
2	0.04124	0.02833	0.05095
3	0.09559	0.00918	0.01395
4	0.13809	0.00556	0.00000
**Total BCRs**	**192354**	**13837**	**3513**

[1]Number of replicates in which a unique BCR is captured

[2]Proportion of total BCRs captured in respective number of replicates in 3'MPLX-amplified samples

[3]Proportion of total BCRs captured in respective number of replicates in 5'RACE-amplified samples

[4]Proportion of total BCRs captured in respective number of replicates in 5'MPLX-amplified samples

Table S3. Primers

Primer name	IGH region	Primer sequence	Methods used
oligo dT	PolyA	TTTTTTTTTTTTTTTTTTTTTTT	5'RACE
JH_BC	J region	TGUCCAGCACGCTUCAGGCUNNNNUNNNNUNNNNCTTACCTGAGGAGACGGTGACC	3'MPLX
JH	J region	CTTACCTGAGGAGACGGTGACC	3'MPLX, 5'RACE, 5'MPLX
VH1-FR1	V region	GGCCTCAGTGAAGGTCTCCTGCAAG	3'MPLX, Isotype
VH2-FR1	V region	GTCTGGTCCTACGCTGGTGAAACCC	3'MPLX, Isotype
VH3-FR1	V region	CTGGGGGGTCCCTGAGACTCTCCTG	3'MPLX, Isotype
VH4-FR1	V region	CTTCGGAGACCCTGTCCCTCACCTG	3'MPLX, Isotype
VH5-FR1	V region	CGGGGAGTCTCTGAACATCTCCTGT	3'MPLX, Isotype
VH6-FR1	V region	TCGCAGACCCTCTCACTCACCTGTG	3'MPLX, Isotype
VH1-FR1_BC	V region	AAGCAGUGGTAUCAACGCAGAGUNNNNUNNNNUNNNNUGGCCTCAGTGAAGGTCTCC TGCAAG	5'MPLX
VH2-FR1_BC	V region	AAGCAGUGGTAUCAACGCAGAGUNNNNUNNNNUNNNNUGTCTGGTCCTACGCTGGTG AAACCC	5'MPLX
VH3-FR1_BC	V region	AAGCAGUGGTAUCAACGCAGAGUNNNNUNNNNUNNNNUCTGGGGGGTCCCTGAGACT CTCCTG	5'MPLX
VH4-FR1_BC	V region	AAGCAGUGGTAUCAACGCAGAGUNNNNUNNNNUNNNNUCTTCGGAGACCCTGTCCCT CACCTG	5'MPLX
VH5-FR1_BC	V region	AAGCAGUGGTAUCAACGCAGAGUNNNNUNNNNUNNNNUCGGGGAGTCTCTGAACATC TCCTGT	5'MPLX
VH6-FR1_BC	V region	AAGCAGUGGTAUCAACGCAGAGUNNNNUNNNNUNNNNUTCGCAGACCCTCTCACTCA CCTGTG	5'MPLX
5'universal	n/a	AAGCAGTGGTATCAACGCA	5'RACE, 5'MPLX
3'universal	n/a	TGUCCAGCACGCTUCAGGC	3'MPLX, Isotype
5'Oligo	n/a	AAGCAGUGGTAUCAACGCAGAGUNNNNUNNNNUNNNNUCTTrGrGrGrG	5'RACE
IGHA	C region	TGTCCAGCACGCTTCAGGCTNNNNTNNNNTNNNNTCAGCGGGAAGACCTTGGGGCTG	Isotype
IGHM	C region	TGTCCAGCACGCTTCAGGCTNNNNTNNNNTNNNNGAGGGGGAAAAGGGTTGGGGCGG	Isotype
IGHD	C region	TGTCCAGCACGCTTCAGGCTNNNNTNNNNTNNNNGATGGGGAACACATCCGGAGCCT	Isotype
IGHE	C region	TGTCCAGCACGCTTCAGGCTNNNNTNNNNTNNNNTCAAGGGGAAGACGGATGGGCTCT GTGT	Isotype
IGHG	C region	TGTCCAGCACGCTTCAGGCTNNNNTNNNNTNNNNGGAAGACCGATGGGCCCTTGGTGG	Isotype

Table S4 Sample filtering information for isotype-resolved sequencing

Patient ID	Days after first sample	Time point	Number of barcoded reads	Number of unique barcodes	Number of Reads after QC filtering (including unique barcodes)	Number of Unique Sequences	Accession number
Healthy 1	0	0	28828	28696	27261	24111	EGAN00001588633
Healthy 2	0	0	163021	161947	153137	132937	EGAN00001588625
Healthy 3	0	0	12444	12434	11817	11296	EGAN00001588631
Healthy 4	0	0	73520	73341	69812	63686	EGAN00001588629
Healthy 5	0	0	63183	62937	58794	53890	EGAN00001588611
Healthy 6	0	0	15053	15039	14356	13022	EGAN00001588634
Healthy 7	0	0	12454	12449	11858	11232	EGAN00001588631
Healthy 8	0	0	13843	13833	13021	12604	EGAN00001588636
Healthy 9	0	0	20242	20228	19219	18263	EGAN00001588634
Healthy 10	0	0	22513	22496	21357	20186	EGAN00001588633
Healthy 11	0	0	24041	24023	22901	20914	EGAN00001588634
Healthy 12*	0	0	28510	28485	27062	26206	EGAN00001588635
Healthy 13	0	0	163021	161947	153137	132937	EGAN00001588625
Healthy 14*	0	0	8626	8622	8123	7908	EGAN00001588635
Healthy 15	0	0	21152	21106	20036	19333	EGAN00001588636
Healthy 16	0	0	54234	54051	51886	44208	EGAN00001588624
Healthy 17	0	0	42484	42408	40610	34804	EGAN00001588614
Healthy 18	0	0	62985	62853	61246	52353	EGAN00001588615
Healthy 19	0	0	68705	67901	64264	51679	EGAN00001588629
CLL 1	0	0	138059	136991	130626	21736	EGAN00001588577
CLL 1	182	1	96852	96415	91701	38031	EGAN00001588587
CLL 2	0	0	160403	158974	149931	20081	EGAN00001588580
CLL 2	28	1	165362	163614	153564	21434	EGAN00001588581
CLL 2	119	2	231594	228742	216170	48759	EGAN00001588585
CLL 3	0	0	193308	191287	184987	24130	EGAN00001588589
CLL 3	255	1	152171	150766	145282	19860	EGAN00001588586
CLL 4	0	0	225594	214141	197183	22312	EGAN00001588582
CLL 4	28	1	218099	213526	199550	24004	EGAN00001588583
CLL 5	0	0	267267	263811	255690	44953	EGAN00001588579
CLL 5	140	1	241262	236692	229067	39245	EGAN00001588584
CLL 6	0	0	210314	208072	204582	29516	EGAN00001588578
CLL 6	151	1	165680	162929	159139	30252	EGAN00001588588
S1_A	n/a		139069	20610	16531	7552	EGAN00001588539
S2_B	n/a		37901	11330	7252	5166	EGAN00001588556
S3_C	n/a		92692	17512	12749	5416	EGAN00001588546
S4_D	n/a		121162	11731	9138	3926	EGAN00001588542

S5_E	n/a		67727	1643	1035	650	EGAN00001588543

*The two samples were pooled together during sequencing and thus have identical accession numbers. Sample-specific tags were added to Illumina adapter tags during the library prep which enabled the sequence deconvolution of the data from the respective two samples.

Table S5 Antibody panels for isotype-specific B cell sorting

Marker	Fluorophore	Laser	Channel	Volume	Clone	Supplier
CD3	V500	Violet	405-525/50	1.25 µL	UCHT1	561416, BD Bioscience
CD4	V500	Violet	405-525/50	1.25 µL	RPA-T4	560768, BD Bioscience
CD14	V500	Violet	405-525/50	2.5 µL	M5E2	561391, BD Bioscience
CD19	BV605	Violet	405-605/12	1.25 µL	SJ25C1	363024, Biolegend
CD27	PE Cy7	Yellow Green	561-780/60	2.5 µL	M-T271	356412, Biolegend
CD38	APC	Red	640-670/14	0.6 µL	HB-7	356606, Biolegend
CXCR4	PE	Yellow Green	561-582/15	0.6 µL	12G5	306506, Biolegend
IgM	FITC	Blue	488-530/30	1.25 µL	MHM-88	314506, Biolegend
IgD	PE-CF594	Yellow Green	561-610/20	1.25 µL	IA6-2	562540, BD Bioscience
IgG	APC-H7	Red	640-780/60	1.25 µL	G18-145	561297, BD Bioscience
R2 medium				86.3 µL		

Table S6 Clinical information for CLL patients.

Patient	Age	Gender	Progression of disease	Stage of disease
CLL 1	67	Male	Stable	A
CLL 2	82	Female	Progressive	B
CLL 3	81	Male	Very slow progressive	A/B
CLL 4	72	Female	Stable	B
CLL 5	80	Male	Slowly progressive	B
CLL 6	63	Male	Stable	A

II. Supplementary figures

(Provided as an additional file)
