# Supplementary material for: Combined Influence of B-Cell Receptor Rearrangement and Somatic Hypermutation on B-Cell Class-Switch Fate in Health and in Chronic Lymphocytic Leukemia
Source: Front Immunol. 2018 Aug 10;9:1784. doi: 10.3389/fimmu.2018.01784 (PMC6095981; doi:10.3389/fimmu.2018.01784)
Supplement: Supplementary file 2 [file Data_Sheet_2.pdf]

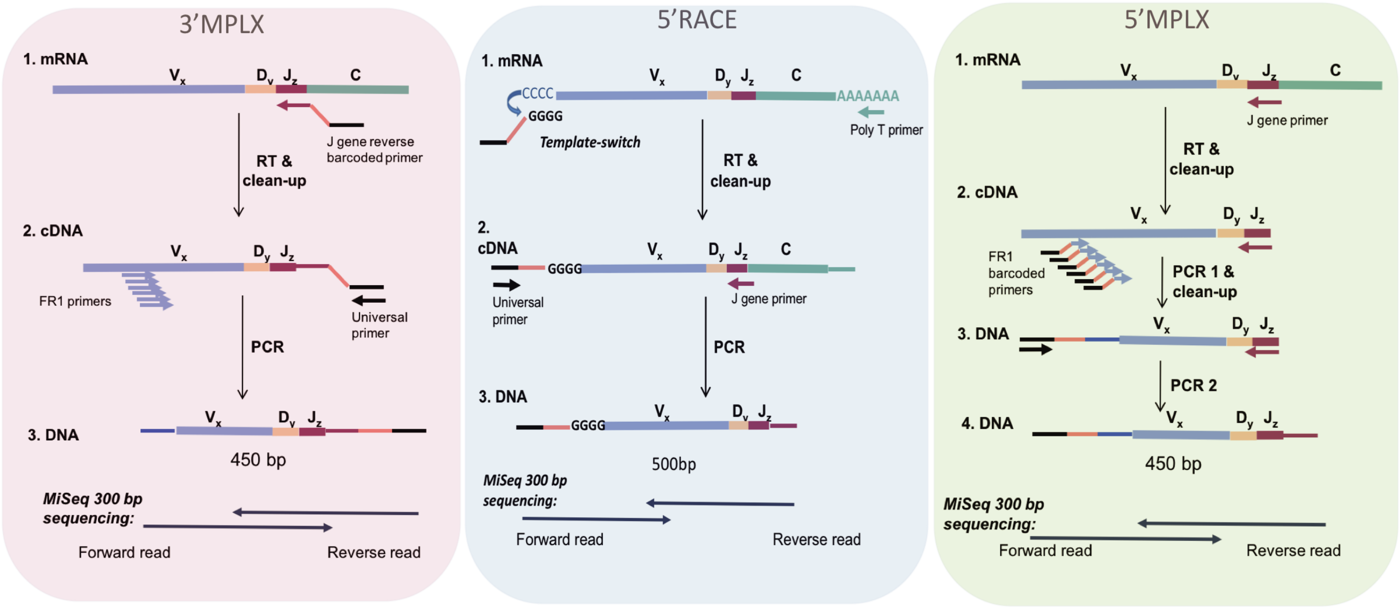

Figure S1. Methods for library preparation using primer IDs

a. 3'Multiplex PCR (3'MPLX) method; 16nt barcodes (5'NNNNTNNNNTNNNNT3') were introduced during reverse transcription (RT) using the reverse *IGHJ*-gene primer; the forward *IGHV* gene mix includes 6 primers for Framework Region 1 (FR1); amplicon size: 400bp; **b.** 5' Multiplex PCR (5'MPLX) method with barcode introduced on each of the 6 *IGHV* gene primers during the first PCR step; Amplicon size: 400-450 bp. **c.** 5'RACE method with a barcode introduced via template-switch during RT; a polyT primer is used for cDNA priming, and a J non-barcoded primer for the PCR step; amplicon size: 550bp;

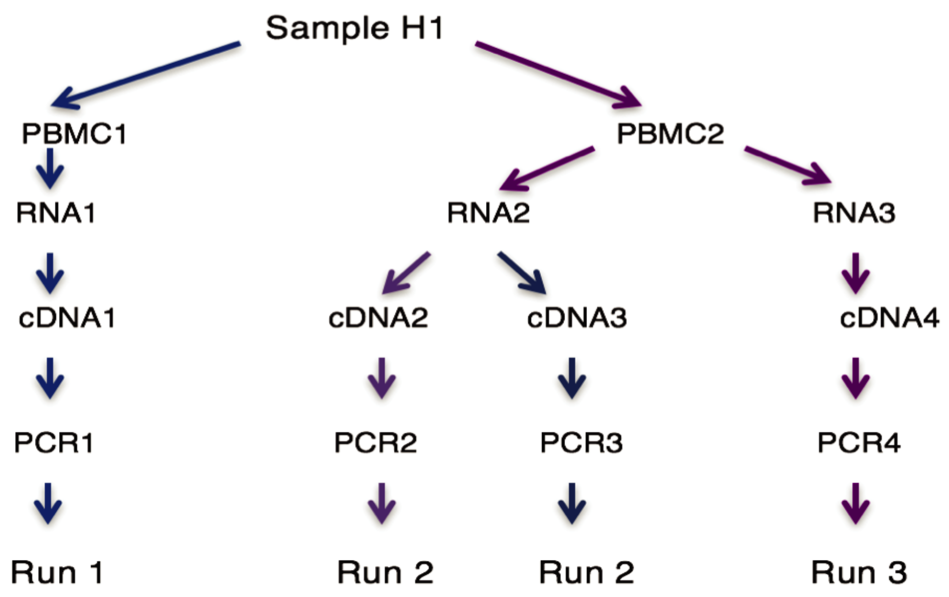

Figure S2. Re-sampling of a healthy PBMC repertoire across biological and technical replicates

Schematic of sample splitting strategy across biological ("PBMC1" and "PBMC2") and technical replicates and the resulting amplified PCR products ("H1_a", "H1_b", "H1_c", "H1_d") used for assessment of reproducibility of library preparation methods

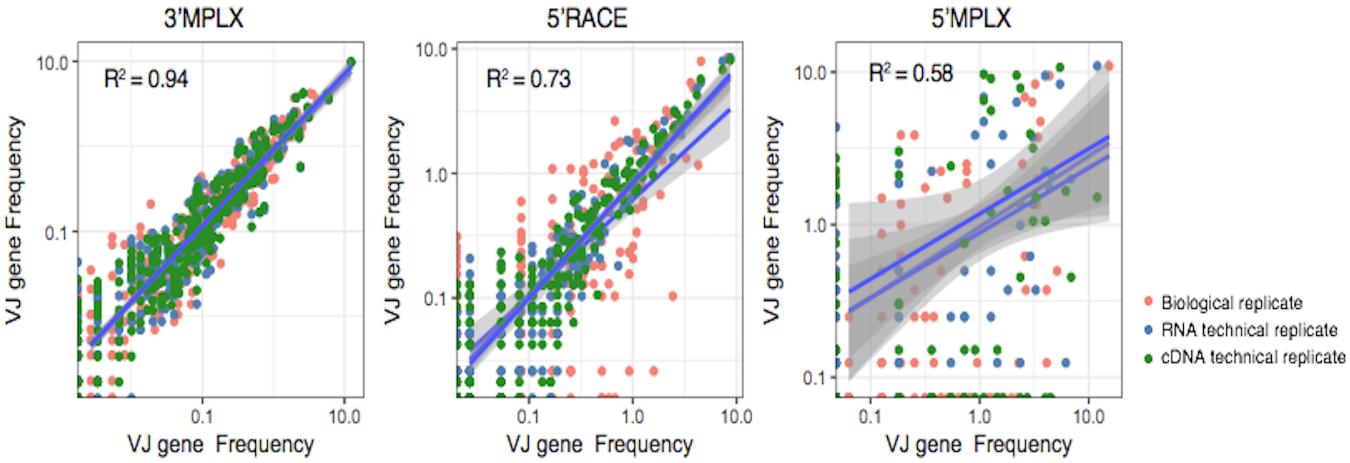

Figure S3. Reproducibility of VJ gene frequency estimates across biological and technical replicates

Pearson correlation of VJ gene frequencies (as % of total repertoire) across the sequenced replicates "H1_a", "H1_b", "H1_c", "H1_d" for each of the three library preparation methods (as shown in figure S2). The plotted biological replicates represent V J gene frequencies of reaction PCR1 (x-axis) vs mean VJ frequencies for reactions PCR2, 3, 4 (y-axis). Technical RNA and cDNA replicates are plotted as 'RNA1' vs 'RNA2', 'RNA1' vs RNA3', 'RNA2 vs RNA3' and 'cDNA1' vs 'cDNA 2', 'cDNA 1' vs cDNA3', 'cDNA2 vs cDNA3'. Gray shading indicates 95% confidence level interval for predictions from a linear model ("lm")

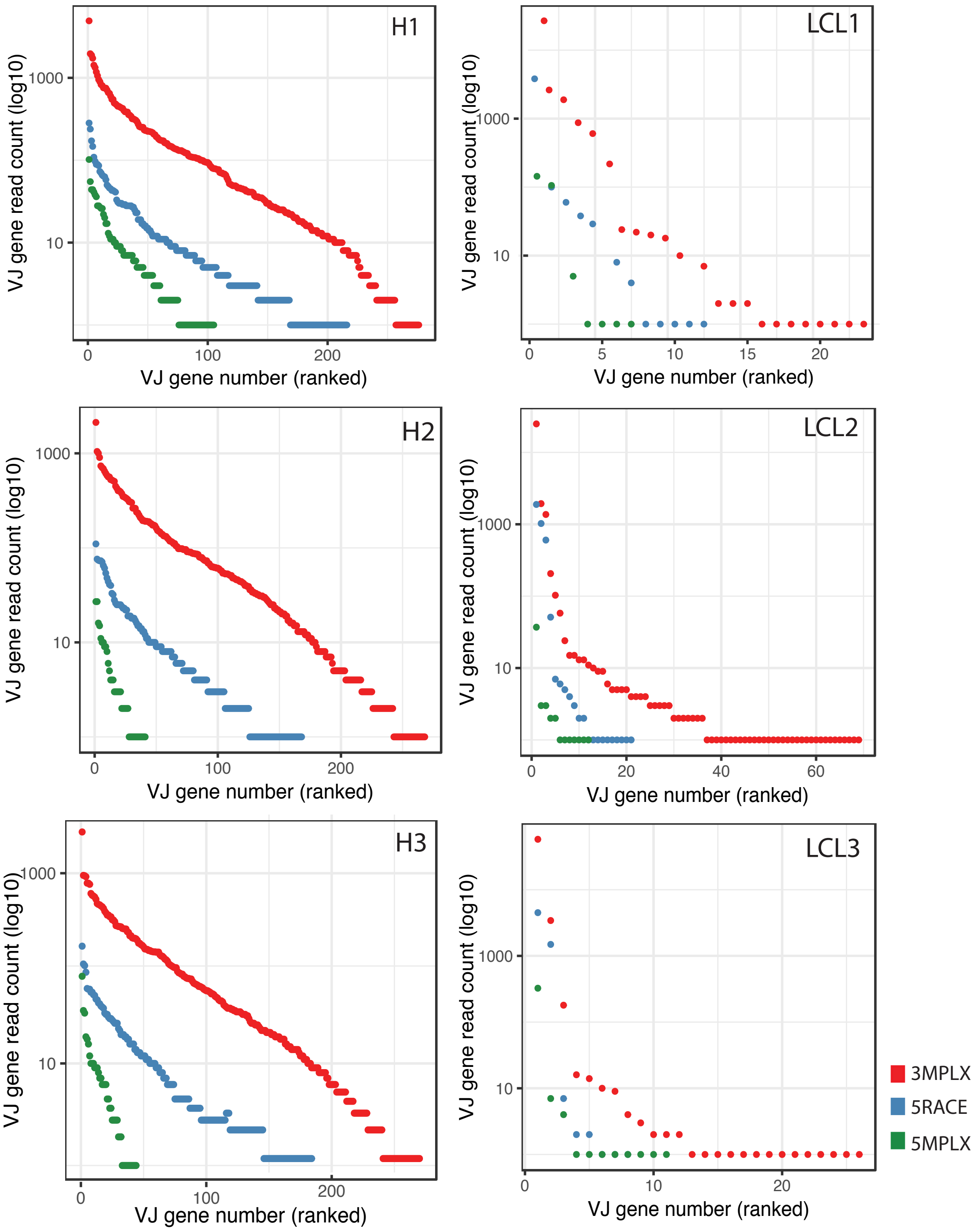

Figure S4. Frequency range of captured VJ genes in PBMC and LCL repertoires

Total read counts representing VJ gene combinations across methods for healthy PBMC samples and LCL samples ordered by descending VJ gene frequency for each method; For sample H1, the mean VJ gene counts across replicates shown in Figure S2 were used

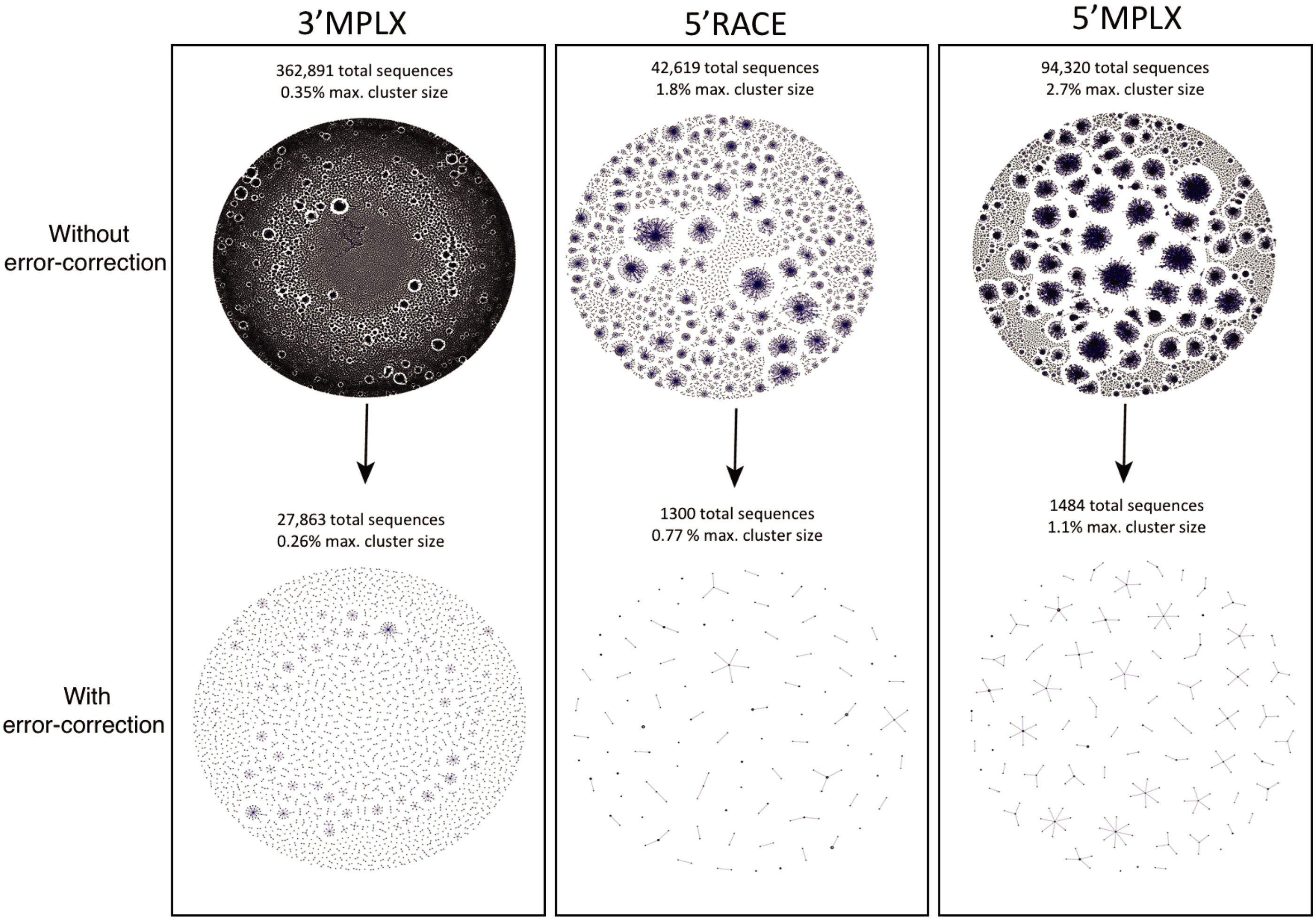

Figure S5. The effect of barcode filtering and error correction across methods

Networks of a representative healthy PBMC sample, H1_a, derived with or without barcode filtering for each of the three used barcode incorporation methods. For non-filtered networks (”Without error correction”), reads were treated as non-barcoded with primers and barcode sequences trimmed before clustering. Networks marked as ‘With error correction’ were generated after the filtering steps described in ‘Methods’.

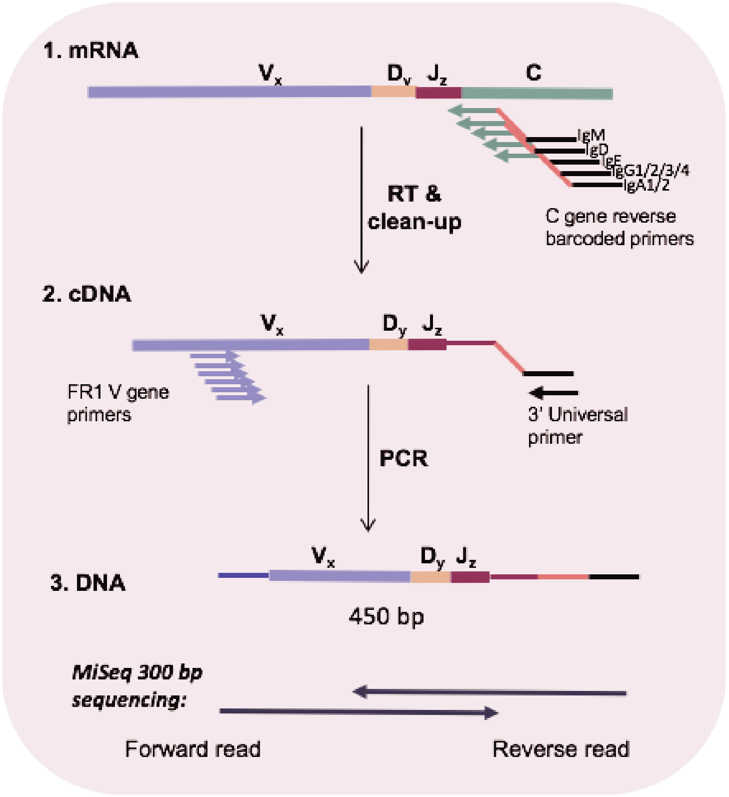

Figure S6. Library preparation steps of isotype-resolved BCR sequencing method

Amplification strategy for simultaneous amplification of immunoglobulin (Ig) variable and constant region genes using 3' multiplex primer set to capture all Ig classes in a single reaction; Each reverse primer has a 15 nt unique molecular identifier (UMI) for correction of sequencing and amplification biases and quantification of BCR abundance. PCR amplification of barcoded cDNA uses a multiplex primer set of 6 *IGHV* FR1-specific primers ad a 5'universal primer for amplification across the UMI. The size of the resulting PCR amplicon is ~450bp and is sequenced by 300bp paired-end MiSeq libraries.

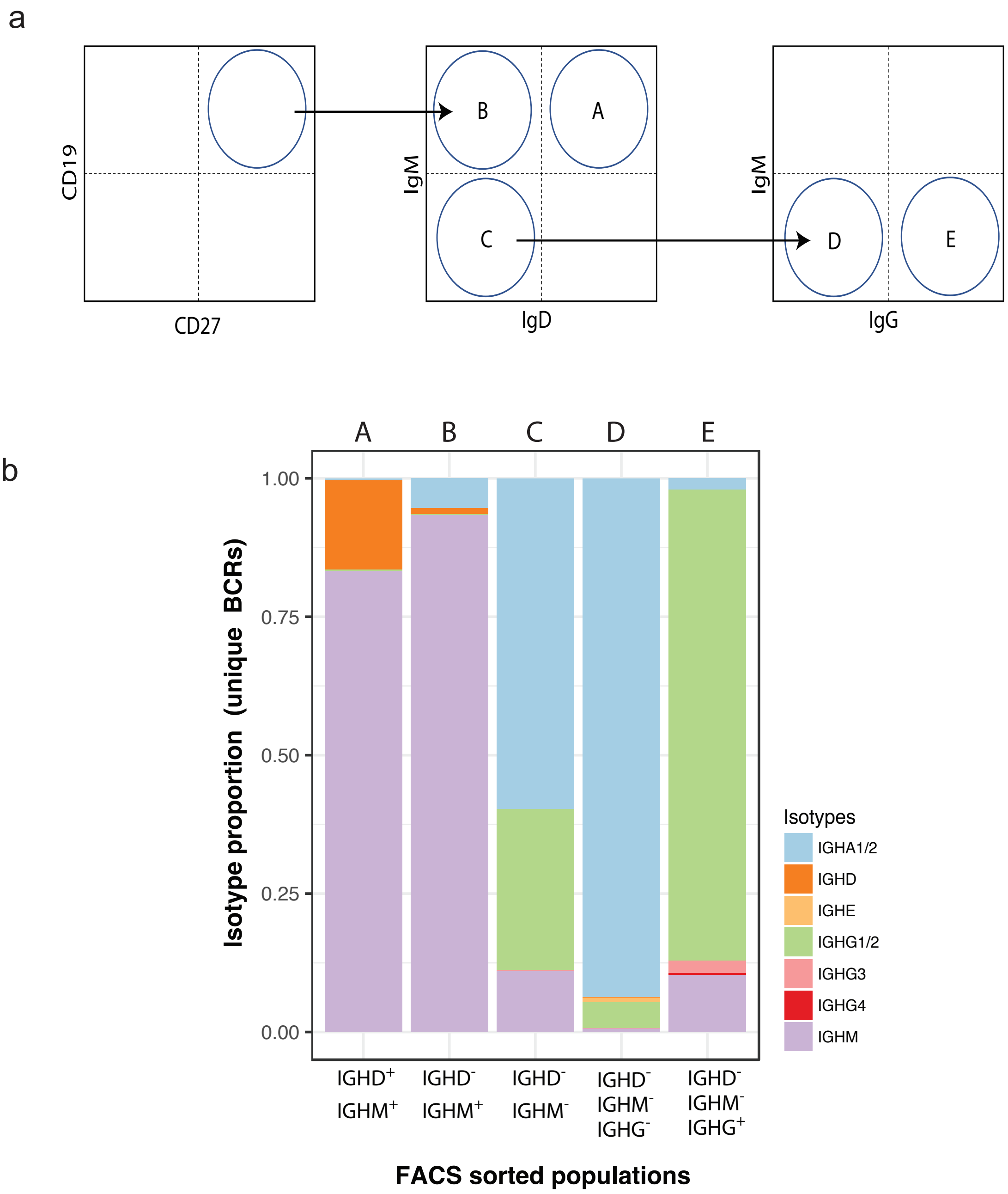

Figure S7. Isotype-resolved sequencing of FACS sorted B memory populations

a. Schematic of FACS sorting strategy of isotype-specific B cell populatins. **b.** Isotype frequency distribution of sorted B-cell populations based on unique BCR sequences per istype. The x-axis labels represents the surface expression of CD19$^+$CD27$^+$ B memory cells from a healthy donor sorted into five isotype-specific populations: CD19$^+$CD27$^+$IGHD$^+$IGHM$^+$, CD19$^+$CD27$^+$IGHD$^-$IGHM$^+$, CD19$^+$CD27$^+$IGHD$^-$IGHM$^-$, CD19$^+$CD27$^+$IGHD$^-$IGHM$^-$IGHG$^-$, CD19$^+$CD27$^+$IGHD$^-$IGHM$^-$IGHG$^+$. RNA from each sorted population was amplified with a mixture of all isotype-specific primers. The relative proportions of individual isotypes were determined by calculating the frequency of unique BCRs from a given isotype as a percentage of the total repertoire for each sorted population.

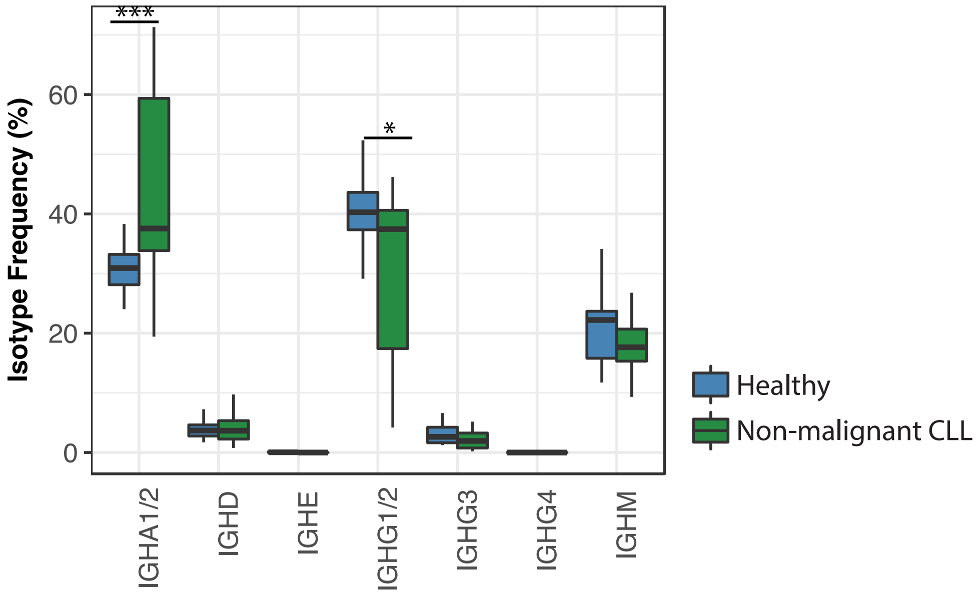

Figure S8. Relative isotype frequency in healthy and non-malignant CLL

Comparison of isotype frequencies as percentage of reads associated with a specific isotype in healthy and in non-malignant CLL repertoires. All BCRs with *IGHV-J* gene identity corresponding to major leukemic clones in CLL patients were also removed from the healthy repertoires for better comparison with the non-malignant CLL repertoires (i.e. CLL repertoires after removal of BCRs with *IGHV-J*genes and/or CDR3 amino acid sequences corresponding to the major CLL clone in each patient). *** - p value < 0.0005; * -p value < 0.05 (Wilcoxon sum ranked text).
